# 晚期非小细胞肺癌免疫治疗超进展的研究进展

**DOI:** 10.3779/j.issn.1009-3419.2021.101.08

**Published:** 2021-04-20

**Authors:** 舒洋 姚, 可鉴 史, 毅 张

**Affiliations:** 100053 北京，首都医科大学宣武医院胸科 Department of Thoracic Surgery, Xuanwu Hospital Capital Medical University, Beijing 100053, China

**Keywords:** 肺肿瘤, 超进展, 免疫检查点抑制剂, 免疫治疗, Lung neoplasms, Hyperprogressive disease, Immune checkpoint inhibitors, Immunotherapy

## Abstract

超进展(hyperprogressive disease, HPD)是一种由免疫检查点抑制剂(immune checkpoint inhibitors, ICIs)治疗导致的进展模式。它表现为肿瘤爆发式生长和极差的患者预后。目前各研究中HPD的定义不尽相同。虽然已经发现HPD与多种临床病理特点和基因类型有关，但目前仍没有可以准确预测它的肿瘤标志物，而且潜在的机制也尚在研究中。本文着眼于晚期非小细胞肺癌，对HPD的研究现状进行综述，以期给采用ICIs药物治疗患者的临床医生提供有益的参考。

## Abstract (en)

Hyperprogressive disease (HPD) is a novel pattern of progression caused by immune checkpoint inhibitors (ICIs). It is characterized by a dramatic tumor surge and is associated with poor clinical outcomes. Up to now, the definition of HPD is still controversial across various studies. Although it has been indicated that HPD has related to multiple clinicopathological features and genetic alterations, it is lack of biomarker to predict its occurrence, and the potential mechanism remains unknown. This review is to summarize current data on HPD specialized in the field of non-small cell lung cancer. And we expect to provide helpful clinical strategies for oncologists using ICIs.

近几年，免疫检查点抑制剂(immune checkpoint inhibitors, ICIs)因其生存优势和良好的耐受性，在驱动基因突变阴性非小细胞肺癌(non-small cell lung cancer, NSCLC)治疗中取得了突破性进展，并因此改变了晚期NSCLC的治疗策略^[1]^，因应用免疫治疗使晚期NSCLC患者5年生存率由4.7%上升到16%^[2]^。

虽然有20%的患者能从程序性死亡因子-1(programmed death-1, PD-1)/程序性死亡因子配体-1(programmed death-ligand 1, PD-L1)抗体的治疗中长期获益^[3, 4]^，可越来越多的研究发现有一部分患者在ICIs治疗的短期内出现肿瘤的加速生长，也就是超进展(hyperprogressive disease, HPD)。晚期NSCLC的HPD发生率为13%-25.7%^[5-8]^，这部分患者的预后很差，成为肿瘤医生临床工作的一个新挑战。本文从HPD的定义、潜在机制和生物标志物的研究现状等方面进行综述。

## 1 HPD定义

目前，国际上HPD定义尚未统一(表 1列出了研究NSCLC免疫治疗超进展的文章)。研究之初，主要采用单位时间内靶病灶最长径之和变异指标的肿瘤生长动力学(tumor growth kinetics, TGK)^[9]^和单位时间内靶病灶肿瘤体积变异指标的肿瘤生长率(tumor growth rate, TGR)^[7, 10]^来定义HPD，这两个指标的计算基于3次影像学评估结果(ICI治疗前3个月-2周; ICI治疗基线和ICI治疗中/后)。

**表 1:** NSCLC患者的HPD研究 Studies of HPD in NSCLC

Author	Incident rate, NSCLC included	Treatment lines	Criteria	Clinical or biological predictors
Kato, 2017^[10]^	38 NSCLC/3 HPD	Anti-PD-1, anti-PD-L1, anti-CTLA4 or other investigational ICIs Different treatment lines	TTF < 2 months, > 50% increase in tumor burden compared with baseline pre-IO imaging, and > 2-fold increase in progression pace	*MDM2*/*MDM4* alteration and EGFR mutation
Kim, 2018^[12]^	17% HPD (37/220 NSCLC)	ICI monotherapy，Line of therapy not clarified	TGK (without details)	None
Kim, ASCO 2019^[8]^	55 (21% HPD) according TGK; 54 (20% HPD) according TGR ratio; 98 (37% HPD) according to TTF < 2 months; Retrospective trial (only NSCLC included)	Anti-PD-1, anti-PD-L1, Different treatment lines	Defined by TGK≥2, TGR ratio≥2 or TTF < 2 months	Higher frequency of severely exhausted (TIGIT^+^PD1^+^) CD8 T-cells and lower frequency of circulating effector memory (CD45RA- CCR7-) CD8 T cells
Ferrara, 2018^[7]^	14% HPD (56/406 NSCLC treated with IO) 5% HPD (3/59 NSCLC treated with chemotherapy)	Anti-PD-1 or anti-PD-L1 as monotherapy or combination with anti-CTLA4 Different treatment lines	Disease progression at the first evaluation and > 50% change in TGR	> 2 metastatic sites before immunotherapy
Kim, 2019^[5]^	14.3% (48/335 NSCLC) had HPD by volumetric assessment and 13.1% (44/335) had HPD as by one-dimensional criteria	Anti-PD-1, anti-PD-L1, Different treatment lines	HPD defined according to one-dimensional or volumetric time-dependent criteria	Volumetric definition of HPD correlated with overall survival; High derived neutrophil-lymphocyte ratio correlated with HPD; 3 (18%) out of 16 HPD patients had LKB1 mutation
Matos Garcia, 2018^[15]^	15% (33/214) HPD (different tumor types included, NSCLC *n*=30)	Anti-PD-1 or anti-PD-L1 as monotherapy or combination Different treatment lines	TTF < 2 months and: (1) increase ≥20% in target tumor burden plus multiple new lesions or (2) increase of ≥40% in target tumor burden compared with baseline	None
Abbar, 2021^[14]^	169 ICI-treated patients included. HPD accounting for 11.3%, 5.7%, 17.0%, 9.6% and 31.7% patients, according to TGR ratio, ΔTGR, TGK, RECIST and TTF definitions	82.2% patients received nivolumab, 10.7% with Pembrolizumab and 7.1% with combination of nivolumab plus ipilimumab. 83.4% patients received the treatment as second-, or subsequent-line settings	The HP incidence rate was calculated according to five definitions (TGR ratio, ΔTGR, TGK, RECIST and TTF)	None
Lo Russo, 2019^[6]^	25.7% (39/152 NSCLC evaluable)	Anti-PD-1 or anti-PD-L1 as monotherapy or combo with anti-CTLA4 Different treatment lines	HPD on the basis of 3 concomitant out of the 5 possible criteria: TTF < 2 months, ECOG PS≥2 during the first 2 months, appearance of at least 2 new lesions in an organ, spread of the disease to a new organ, increase ≥50% in the sum of target lesions major diameters	Clustered macrophages with epithelioid morphology and co-localization of CD163, PD-L1, and CD33 markers (defined as complete phenotype)
HPD: hyperprogressive disease; NSCLC: non-small cell lung cancer; ICIs: immune checkpoint inhibitors; TTF: time to treatment failure; TGR: tumor growth rate; TGK: tumor growth kinetic; ECOG PS: Eastern Cooperative Oncology Group performance status; RECIST: Response Evaluation Criteria in Solid Tumor; PD-L1: programmed death ligand 1; PD-1: programmed cell death 1				

### 1.1 基于TGK的HPD定义

由Ferte^[11]^于2014年首次报道了TGR的计算公式，以评估单位时间内肿瘤体积增长的情况。TGR可对肿瘤进行动态、定量评估，有助于临床医生判定药物对肿瘤的控制。2017年Gustave Roussy团队^[10]^首次报道了接受ICI治疗的肿瘤患者出现HPD的情况。他们在一项Ⅰ期临床试验中发现131例接受ICI治疗的患者，12例(9%)出现了肿瘤加速生长。该研究对HPD的定义为：由实体瘤疗效评价标准(Response Evaluation Criteria in Solid Tumor, RECIST)1.1判定为疾病进展(progression disease, PD)的患者，治疗后TGR/治疗前TGR≥2。并且发现HPD与肿瘤类型和初始肿瘤负荷量无关，而与高龄和预后差密切相关(*P* < 0.05)，该研究并没有对NSCLC患者HPD的情况进行单独说明。Kim等^[12]^通过TGR来定义HPD，220例接受ICI单药治疗的晚期NSCLC患者的发生率为17%，且HPD与更差的无进展生存时间(progression-free survival, PFS)和总生存时间(overall survival, OS)明显相关，而与性别、吸烟史、PD-L1表达水平、既往化疗治疗线数、放疗、更高的基线肿瘤负荷无关。Ferrara等^[7]^回顾性分析了8个法国治疗中心接受过ICI治疗的406例NSCLC患者，HPD率为13.8%(56/406)。他们的HPD定义为与ICI治疗前的TGR相比，治疗中TGR增加超过50%。HPD与ICI治疗前转移部位的数量相关，HPD患者的OS明显差于非HPD的PD患者。这一研究还包括了单纯化疗组作为对照，结果发现多线治疗后的单药化疗患者的HPD率为5%(3/59)。

### 1.2 基于TGK的HPD定义

TGK最初由Tourneau提出^[13]^，接受分子靶向治疗患者在判断疗效时，其作为RECIST标准的补充。Saada-Bouzid等^[9]^将HPD定义为TGK比值(免疫治疗后的TGK与治疗前的TGK比值)≥2。一项韩国研究^[8]^纳入了263例PD-1/PD-L1治疗的复发和(或)转移性NSCLC，分别根据治疗失败时间(time to treatment failure, TTF)、TGR和TGK的标准来判定HPD，发生率分别为98例(37.3%)、54例(20.5%)和55例(20.9%)。由TGK和TGR标准判定的HPD与非HPD的传统PD相比，有明显更差的PFS(HR=4.619; 95%CI: 2.868-7.440)和OS(HR=5.079, 95%CI: 3.136-8.226)。而2020年Abbar等^[14]^对169例应用ICIs治疗的NSCLC患者，从TGR比率、ΔTGR、TGK、RECIST和TTF五个方面定义了HPD，发生率分别为11.3%、5.7%、17.0%、9.6%和31.7%，不同定义标准的不一致性高达29%-77%，只有TTF定义的HPD患者比非HPD患者存在有统计学意义的更差OS。由此看出不同研究的结果存在明显的差异还需要进行更多的研究。

### 1.3 TGR或TGK定义HPD存在的问题

只根据TGR或TGK来定义HPD存在以下问题：首先，临床中的患者，尤其是ICI作为NSCLC一线治疗的患者，很难完成计算TGR或TGK所需要的三次影像学检查。其次，RECIST和修改后的评估免疫治疗的RECIST(modified RECIST 1.1 for immune based therapeutics, iRECIST)标准只是评估肿瘤大小的变化，而没有重视非靶病灶如胸腹腔积液、癌性淋巴管炎、骨转移以及10 mm以下的病灶。最后，患者的临床表现和生活质量也没有考虑在内。

### 1.4 其他HPD定义

还有研究者采用了没有纳入TGR或TGK的HPD定义。Matos等^[15]^将HPD定义为TTF < 2个月，可测量病灶至少增加10 mm，目标肿瘤负荷比基线增加40%或增加20%以上且出现多个新病灶。在241例接受ICI治疗的实体瘤患者中(14%为肺癌)，15%(33例)出现HPD。HPD与更差的OS密切相关，但与ICI药物类型、年龄、转移部位或肿瘤类型无关。Lo Russo等^[6]^认为HPD应至少符合以下5项标准中的3项：①TTF < 2个月; ②在免疫治疗最初的2个月内美国东部肿瘤协作组(Eastern Cooperative Oncology Group, ECOG)日常体能状态(performance status, PS)恶化，评分≥2分; ③基线和第一次肿瘤评估之间靶病灶最大径总和增加≥50%; ④基线和第一次肿瘤评估之间转移到新器官; ⑤基线和第一次肿瘤评估之间一个器官至少出现2个新病变。在152例ICI治疗的NSCLC患者中，39例(25.7%)出现HPD，OS明显降低(4.4个月，95%CI：3.4-5.4)。另外，2018年欧洲肿瘤学年会上报道的Ⅲ期临床研究(OAK)的一项亚组分析^[16]^定义了快速进展(fast progression, FP)患者，治疗开始后6周靶病灶最长径之和增长≥ 50%或者未接受肿瘤评估的患者在12周内死亡。使用这一定义发现接受阿特珠单抗治疗患者的FP率与化疗患者的相似。然而，一项大规模回顾性研究^[17]^发现FP与HPD的重叠部分很小。这一研究还发现FP与患者基线PS高显著相关，而且与HPD患者相比，FP患者的OS更差，这说明FP可能是一种与治疗疗效差相关的负性疗效预测因子，而非用于鉴别ICI特异性相关的肿瘤生长加速的因子。

## 2 HPD机制的研究进展

HPD发展的机制目前尚未明确。可能的机制包括T调节细胞的扩增、T效应细胞的耗竭、肿瘤免疫的调节、局部异常炎性反应和肿瘤通路的激活等^[18]^。

### 2.1 非特异性免疫细胞的作用

Lo Russo等^[6]^在出现HPD的NSCLC患者中，发现了具有上皮表型的巨噬细胞簇，且同时表达CD163、PD-L1和CD33(定义为完全表型)。此外，患者来源的杂交动物模型中发现经由Fc-Fc受体信号通路重组M2免疫抑制巨噬细胞，抗PD-1抗体可能驱动HPD的发生。

其他非特异性免疫细胞如中性粒细胞也在HPD的过程中发挥作用。接受ICI治疗的NSCLC患者，高衍生粒淋比(derived neutrophil-lymphocyte ratio, dNLR)与预后差^[19, 20]^和HPD的发生^[5]^相关。鉴于dNLR可能也是化疗患者疗效的预测因子，那么特异性循环中性粒细胞亚型可能与肿瘤的加速生长有关^[21]^。还发现ICI治疗后快速进展的NSCLC患者，其外周血中，幼稚中性粒细胞(CD16低)明显增多^[22]^。同时，动物体内试验也对中性粒细胞在ICI相关HPD中所起作用进行了探索。表达人源IL-17A的肺腺癌移植小鼠对PD-1抑制剂耐药，并出现广泛的肿瘤内中性粒细胞浸润; 同时发现，如果阻断IL-6或耗竭中性粒细胞能导致有效的免疫反应^[23]^。

### 2.2 T效应细胞的耗竭

HPD也能通过免疫抑制T细胞进行调节。与非HPD的NSCLC患者相比，发现HPD患者在使用ICI之前，循环效应记忆CD8^+^ T细胞(CCR7^-^CD45RA^-^)更低，而严重的耗竭CD8^+^ T细胞(TIGIT^+^PD1^+^)更高^[8]^。

### 2.3 特定基因的异常表达

特定的基因表达异常(比如*EGFR*、*MDM2*、*LKB1*)与肿瘤免疫微环境息息相关，而后者有助于ICI治疗相关的肿瘤生长加速。在16例NSCLC患者中发现3例(18%)*LKB1*和*KRAS*双突变者出现HPD^[5]^。在动物实验中发现*LKB1*突变与中性粒细胞聚集相关和肿瘤细胞上PD-L1低表达相关^[24]^。

### 2.4 信号通路的激活

HPD还可能由肿瘤生长信号通路的激活所致。食管和肾细胞癌患者在ICI治疗后出现HPD，在肿瘤中发现肿瘤抑制基因如TSC2和VHL的散发突变，同时还发现了包括IGF-1、ERK/M APK、PI3K/AKT等多条肿瘤信号通路转录的上调^[25]^。PD-1轴抑制了T细胞淋巴瘤的增殖(通过下调PI3K通路)，说明HPD的细胞自主机制源于PD-1对肿瘤细胞的直接抑制^[26]^。

## 3 HPD相关标志物的研究进展

多项研究^[7, 8, 10]^证实HPD患者预后极差，因此，寻找到可以预测ICI治疗中/后出现HPD的标志物尤为重要。HPD风险分层分析是一个挑战，

### 3.1 临床病理特点

因为通常难以获得随机试验中HPD评价所需要的基线前电子计算机断层扫描(Computed Tomography, CT)扫描结果。在一些回顾性研究中发现HPD可能与多种临床病理特点相关，包括升高的乳酸脱氢酶(serum lactate dehydrogenase, LDH)，两个以上的转移部位(尤其是肝转移患者)，升高的dNLR，高于正常的血小板绝对值以及高皇家马斯登医院预后评分结果^[27-30]^，同时也发现与既往治疗、分期和PS评分没有明显相关性^[7, 10, 18]^。Petrova等^[31]^的回顾性分析发现升高的免疫治疗前dNLR和肌少症(定义为：治疗前后腰大肌变化率大于10%)与HPD的发生有显著相关性。Ku等^[32]^发现HPD患者具有高dNLR，瘤内B细胞密度与HPD的发生显著相关，而与T细胞和巨噬细胞密度无关。

### 3.2 基因异常表型

基因异常表型如MDM2/MDM4扩增和EGFR突变或STK11与KRAS的双突变可能预示HPD的高风险^[5, 33]^。基因多态性如rs2282055(*PD-L1*)和rs1870377(*VEGFR2*)预示着HPD发生的高风险^[34]^。作为P53的主要负性调节子的*MDM2/4*基因，一旦出现扩增会对T细胞介导的肿瘤杀伤产生抵抗，因此ICIs的疗效差^[35]^。*EGFR*突变与PD-L1低表达和肿瘤微环境中T细胞失活相关，因此这类NSCLC患者对ICIs的有效率低^[36]^。然而，*EGFR*突变在HPD中的作用还需要进一步的研究。

这些预测标志物在不同研究中的结论可能相互矛盾，需要前瞻性临床试验来对它们的临床可行性进行验证。比如，最近一篇个案报道的HPD患者^[37]^，治疗后TGR/治疗前TGR升高，但并没有*MDM2*或*EGFR*基因突变，而有*ERBB2*基因异常和PD-L1高表达。尽管多项研究中，肿瘤PD-L1的表达与HPD相关性的结论不一致^[6, 28]^，但是阐述二者潜在相关性以及发现更多的标志物是非常重要的，只有这样才可以把患者进行分层，筛选出计划一线ICI治疗而有HPD高风险的患者。

### 3.3 肿瘤微环境

肿瘤微环境中，既有的抗肿瘤免疫状态也可能对HPD起作用。出现HPD的NSCLC患者据报道^[5]^有循环效应/记忆CD8^+^ T细胞(CCR7^-^CD45RA^-^)耗竭和更多肿瘤反应性CD8^+^ T细胞(TIGIT^+^PD-1^+^)。一项监测T细胞活性的研究^[38]^发现，在第一周期治疗后，外周血CD28^-^CD4^+^淋巴细胞明显增高，借此可以对接受ICIs治疗的NSCLC患者进行HPD的早期诊断。Kim等^[39]^发现HPD患者的治疗前CD8^+^ T细胞中CD39^+^细胞比例明显高于非HPD者。这种非侵袭性且操作简便的血清学标志物检测可能有助于HPD的诊断。Kim等^[40]^观察到晚期NSCLC患者免疫治疗后肿瘤微环境中CD8^+^/PD-1^+^ TIL更少，M2吞噬细胞增多与HPD的发生密切相关。

### 3.4 肿瘤瘤周影像学特点

基线CT扫描的肿瘤瘤周影像学特点也可以预测HPD。Vaidya等^[41]^的研究发现患者PD-(L)1单药治疗前后CT影像学的特点(包括瘤周5 mm-10 mm的特征和两个肿瘤血管相关的弯曲特点)是HPD的预测因子。一项纳入50例转移性或复发性NSCLC的前瞻性研究^[28]^，患者均接受^18^F-FDG正电子发射断层扫描(positron emission tomography, PET)/CT和胸部加强CT，结果发现HPD与基线肿瘤负荷的高代谢活性显著相关。尽管这些发现还需要更大规模的研究进行验证，但是基于影像学的HPD预测方式是引人注目的，因为这是一种更易于在日常工作中应用的方法。

## 4 免疫治疗与其他抗肿瘤治疗HPD的差异

HPD到底是一种免疫治疗相关的现象还是肿瘤生长的自然进程，目前尚无法明确。在一些人肿瘤杂交动物模型中，发现随着时间的推移，肿瘤出现指数型生长和基因的持续变化^[42, 43]^。为了更客观地回答这个问题，需要设计比较免疫治疗与安慰剂的前瞻对照临床研究。因这样的试验可能不符合伦理，有些研究者把化疗或靶向治疗作为对照组。化疗或靶向治疗的HPD或FR率在5.1%-13.5%^[7, 44]^，说明基线后的快速进展并不是ICIs独有的。有研究^[7]^报道了化疗HPD患者的OS与非HPD进展者没有显著差异[*P*=0.60; 4.5个月(95%CI：2.5-6.5) *vs* 3.9个月(95%CI：2.7-6.9)]。Matos等^[44]^的研究对比了ICIs与靶向治疗的HPD，168例采用TGR标准判定的靶向治疗患者中有12例为HPD，不同治疗组里，HPD患者的OS与非HPD进展者没有显著性差异。现有证据说明HPD并非免疫治疗独有的现象，只是与其他治疗相比，ICI治疗患者的HPD更有临床意义，对患者预后的影响更大。

## 5 HPD的治疗策略

复发性NSCLC患者接受ICIs治疗的早期随机对照Ⅲ期试验发现在早期试验节点，与化疗相比，ICIs治疗患者的死亡风险更高，原因之一就是HPD^[45]^。有趣的是，在随后ICIs联合化疗的临床研究中发现，在联合治疗的初期就能看到患者明显获益，在Checkmate227试验^[46]^中，PD-L1表达 < 1%的亚组，在治疗的第3个月，纳武单抗联合化疗患者的进展风险比纳武单抗联合伊匹单抗低。通过潜在的不同通路，化疗可能有助于防止患者出现ICI耐药和HPD，这值得进一步的研究。化疗除了对肿瘤细胞的细胞毒作用，还能通过多种不同的机制抑制骨髓来源的抑制性细胞; 因此，当化疗与PD-1抑制剂联合使用时，二者可能有协同作用。还有研究^[47]^发现免疫治疗序贯化疗的疗效优于化疗序贯免疫治疗。由此推论，HPD高风险患者可能从化疗与ICIs的联合治疗或在ICI治疗之后迅速调整成化疗中获益。

ICIs另一个独特的“进展”方式是假进展。一项针对ICIs治疗后NSCLC患者的回顾性研究^[48]^发现RECIST标准定义为PD的患者中，8.3%后证实为部分缓解(partial response, PR)。一部分假进展与目前使用的HPD标准有重叠的部分，这就使精确的肿瘤评估变得困难重重。Ferrara等^[7]^研究发现，6例假进展的患者在第一次肿瘤评估时被判断为HPD。新辅助ICIs治疗进一步使我们认识到影像学结果和肿瘤状态的不一致性，比如术前CT显示肿瘤增大，而术后病理显示治疗有效^[49]^。因HPD患者预后极差，现阶段，尽早发现HPD和迅速用化疗替换ICIs是治疗的重中之重。HPD发生率远远超过了假进展率(1%-9%)^[50]^，对疑似假进展的患者必须做到更为严格的影像学评估和更为密切的影像学监测。ICI治疗中的肿瘤评估应在初始治疗的第6周-12周进行，患者影像学显示为进展但病情稳定，可以继续ICI治疗，但必须在4周-8周内接受确认性影像学评估。如果患者第一次影像学评估为PD且同时病情恶化要考虑到HPD的可能。HPD是ICIs治疗的一种凶险的并发症，应该在患者进行治疗之前就告知患者。目前尚无预测HPD的标志物，做好患教工作，让患者在临床访视时告诉医生自己发生的任何病情变化或新的症状。一旦确诊为HPD，应立刻停止免疫治疗，换成另一种完全不同抗肿瘤治疗。临床医生同时应认识到ICI治疗失败后的后线治疗很可能无效^[51]^，而且大部分HPD患者根本没有接受后线治疗的机会^[8, 47]^。

## 6 结论

虽然近几年来，HPD得到越来越多研究者和临床医师的关注，但在诊断标准、发展机制、预测指标和治疗策略中仍有很多未解的问题。大部分HPD患者根本没有后续治疗的机会，尤其值得临床医师在临床工作中保持警惕，及时对这一免疫治疗并发症作出判断。因此，需要尽快在HPD诊断上达成共识，才可能在此基础上进行随机对照研究。同时，对HPD机制和预测标志物的研究也极为重要，只有通过对HPD机制的深入理解，才可能研制出能真正“切断”HPD的新药。
